# Comparison of active treatments for impaired glucose regulation: a Salford Royal Foundation Trust and Hitachi collaboration (CATFISH): study protocol for a randomized controlled trial

**DOI:** 10.1186/s13063-016-1519-6

**Published:** 2016-08-26

**Authors:** Peter A. Coventry, Peter Bower, Amy Blakemore, Liz Baker, Mark Hann, Angela Paisley, Charlotte Renwick, Jinshuo Li, Atushi Ugajin, Martin Gibson

**Affiliations:** 1 Mental Health and Addiction Research Group, Department of Health Sciences, University of York, York, YO10 5DD UK; 2 NIHR School for Primary Care Research and Manchester Academic Health Science Centre, University of Manchester, Manchester, M13 9PL UK; 3 Information Systems Group, Hitachi Europe Limited, London, NW1 5DH UK; 4 Centre for Biostatistics and Manchester Academic Health Science Centre, University of Manchester, Manchester, M13 9PL UK; 5 Salford Royal NHS Foundation Trust, Salford, M6 8HD UK

## Abstract

**Background:**

Diabetes is highly prevalent and contributes to significant morbidity and mortality worldwide. Behaviour change interventions that target health and lifestyle factors associated with the onset of diabetes can delay progression to diabetes, but many approaches rely on intensive one-to-one contact by specialists. Health coaching is an approach based on motivational interviewing that can potentially deliver behaviour change interventions by non-specialists at a larger scale. This trial protocol describes a randomized controlled trial (CATFISH) that tests whether a web-enhanced telephone health coaching intervention (IGR3) is more acceptable and efficient than a telephone-only health coaching intervention (IGR2) for people with prediabetes (impaired glucose regulation).

**Methods:**

CATFISH is a two-parallel group, single-centre individually randomized controlled trial. Eligible participants are patients aged ≥18 years with impaired glucose regulation (HbA1c concentration between 42 and 47 mmol/mol), have access to a telephone and home internet and have been referred to an existing telephone health coaching service at Salford Royal NHS Foundation Trust, Salford, UK. Participants who give written informed consent will be randomized remotely (via a clinical trials unit) to either the existing pathway (IGR2) or the new web-enhanced pathway (IGR3) for 9 months. The primary outcome measure is patient acceptability at 9 months, determined using the Client Satisfaction Questionnaire. Secondary outcome measures at 9 months are: cost of delivery of IGR2 and IGR3, mental health, quality of life, patient activation, self-management, weight (kg), HbA1c concentration, and body mass index. All outcome measures will be analyzed on an intention-to-treat basis. A qualitative process evaluation will explore the experiences of participants and providers with a focus on understanding usability of interventions, mechanisms of behaviour change, and impact of context on delivery and user acceptability. Qualitative data will be analyzed using Framework.

**Discussion:**

The CATFISH trial will provide a pragmatic assessment of whether a web-based information technology platform can enhance acceptability of a telephone health coaching intervention for people with prediabetes. The data will prove critical in understanding the role of web applications to improve engagement with evidence-based approaches to preventing diabetes.

**Trial registration:**

ISRCTN16534814. Registered on 7 February 2016.

**Electronic supplementary material:**

The online version of this article (doi:10.1186/s13063-016-1519-6) contains supplementary material, which is available to authorized users.

## Background

Diabetes is a long-term condition characterized by hyperglycaemia in the presence of defects of insulin secretion or insulin action, or both, and is a major cause of morbidity and premature mortality globally [1]. At present, 3.4 million adults in the UK are diagnosed with diabetes, the majority with type 2 diabetes [2]. The damaging effects of uncontrolled hyperglycaemia can cause macrovascular complications (coronary artery disease, peripheral arterial disease and stroke) and microvascular complications (diabetic nephropathy [kidney disease], neuropathy [nerve damage], which can lead to non-traumatic lower limb amputations, and retinopathy, which can lead to blindness) [3]. Altogether, the impact of diabetes is thus significant, with serious implications for health and quality of life, and costs to health care systems. In England, the direct cost to the National Health Service (NHS) of treating type 2 diabetes is approximately £8.8 billion annually, with a further £13 billion associated with indirect costs; these costs are estimated to rise to £15.1 and £20.5 billion, respectively, by 2035–6 [4].

Obesity, physical inactivity and diet are among key risk factors for type 2 diabetes. Weight gain and obesity are especially implicated in the onset of type 2 diabetes. Obese women are nearly 13 times more likely to develop type 2 diabetes than non-obese women; obese men are over 5 times as likely to develop type 2 diabetes [5]. Furthermore, a 1 kg/m^2^ increase in body mass index increases the risk of impaired fasting glucose by 9.5 % [6]. Impaired fasting glucose or impaired glucose tolerance indicate impaired glucose regulation, which is a condition where blood glucose levels are raised, but the levels are insufficient to meet current thresholds for a clinical diagnosis of type 2 diabetes. Impaired fasting glucose is associated with a raised hepatic glucose output, whereas impaired glucose tolerance is associated with peripheral insulin resistance. There is strong and consistent evidence that people with impaired fasting glucose or impaired glucose tolerance have between a 6- and 12-fold risk of developing diabetes, compared with people without, and both are risk factors for fatal and non-fatal cardiovascular events [7].

However, it is well established that lifestyle interventions that target modifiable risk factors such as weight and physical activity can prevent the onset of diabetes in people with impaired glucose regulation. A systematic review of 36 trials showed that diabetes prevention programmes that included diet or physical activity interventions can significantly reduce progression to type 2 diabetes and reduce weight and glucose at 12–18 months, compared with usual care [8]. As such, identification of people with impaired glucose regulation and intervention with lifestyle-change programmes presents significant opportunities for reducing the future incidence of type 2 diabetes. The delivery of these behaviour change interventions is central to guidance from the National Institute for Health and Care Excellence (NICE) on prevention of diabetes in high-risk groups, including people with impaired glucose regulation. However, the delivery of NICE-recommended diabetes prevention programmes is contingent on the availability of specialist staff to provide intensive interventions to relatively small numbers of people over 9 to 18 months. As articulated by the NHS National Diabetes Prevention Programme, the challenge remains to scale up and rapidly roll out evidence-based behaviour change interventions to ensure that more people at risk of diabetes can access to such interventions, but without compromising quality.

### Health coaching

A model of care that has potential to achieve diabetes prevention at a large scale through effective behaviour change is ‘health coaching’. This is a relatively new approach and variously defined but common to this approach is an emphasis on health education and health promotion via patient-centred coaching based on motivational interviewing to improve health outcomes [9].

The increasing adoption of telephone and mobile technologies among patients, and the possibility of delivering care in efficient and flexible ways, has led to significant interest in the potential of telephone health coaching which involves:

a regular series of phone calls between patient and health professional… to provide support and encouragement to the patient, and promote healthy behaviours such as treatment control, healthy diet, physical activity and mobility, rehabilitation, and good mental health [10]

However, current evidence of effectiveness is mixed. A systematic review of 13 randomized controlled trials or quasi-experimental studies showed that, in 11 studies, telephone, internet or a combination of telephone, face-to-face, internet or email health coaching can effectively improve physical and mental health, promote healthy behaviours and increase social support among people with long-term conditions [11]. Other reviews have similarly identified a number of effective models, although the important ‘active ingredients’ are not clear [12, 13]. Moreover, much of the evidence is derived from trials conducted in the USA and there is uncertainty about the benefits of health coaching in the UK. A recent evaluation of the nurse-led Birmingham OwnHealth telephone health coaching service for people with long-term conditions (including diabetes) did not find reductions in health service utilization or cost over 12 months [14]. By contrast, a UK trial of telephone support from non-clinical telecare staff (backed up by diabetes specialist nurses) did show significant improvements in glycaemic control in people with type 2 diabetes, compared with usual care [15]. This intervention, now known as Diabetes Care Call, has recently been adapted for use in people with impaired glucose tolerance and impaired glucose regulation. A pilot evaluation (*n* = 44) of the Care Call intervention in people with impaired glucose tolerance showed reductions in weight (2.81 kg, 95 % confidence interval 1.2–4.42), body mass index (1.06 kg/m^2^, 95 % confidence interval 0.49–1.63), and fasting blood glucose (0.29 mmol/l, 95 % confidence interval 0.07–0.51) 1 year after the intervention [16]. Similar outcomes 12 months after the intervention were achieved in a pilot evaluation of Care Call in people with impaired glucose regulation who were offered either a telephone-only or a telephone plus group education pathway [17]. While the findings of these pilot studies are limited by the absence of a control group, they offer proof of concept that telephone health coaching can translate to people with impaired glucose regulation to promote positive and sustained lifestyle changes to prevent type 2 diabetes.

The Impaired Glucose Regulation Care Call intervention has recently been enhanced, with greater use of web-based materials and electronic transfer of patient data, with a view to making the service more engaging for patients, and the provision of care more efficient for providers. The web plus telephone health coaching intervention has been developed by NorthWest EHealth in partnership with Hitachi Europe Ltd. Patient engagement is critical to the success of health promotion interventions and frequent, real-time communication and feedback are key to behaviour change and empowering patients to manage their behaviour [18, 19]. Information technology (IT) platforms, such as desktop applications, mobile short message service (SMS) and internet-based interventions are increasingly used to support and enhance patient engagement in self-management programmes. There is partial evidence that e-health interventions, described as second-generation interactive computerized interventions, can lead to positive improvements in physical activity and diet in people drawn from community and health settings [20] and can also support diabetes self-management tasks [21]. However, the evidence in favour of using IT interventions to support behaviour change is equivocal and few studies have assessed whether satisfaction and usability lead to better engagement and less costly delivery [22].

The addition of an IT platform within the Impaired Glucose Regulation Care Call service might lead to significant advantages in patient uptake and engagement, as well as freed human resources for the provider, which could ultimately improve the clinical effectiveness and cost-effectiveness of the service. However, there is a need to conduct an assessment of satisfaction, usability and cost of delivery of the new web-enabled telephone health coaching service (known as IGR3). This trial will therefore compare user experience and cost of delivery of IGR3 with the existing telephone-only health coaching service (known as IGR2). Further study into the potential impact of the IGR3 model on the clinical and cost effective aspects will then be planned.

## Methods/Design

### Trial design

This trial protocol is written in accordance with standardized reporting guidance from SPIRIT (see Additional file 1) [23, 24].

This trial is a pragmatic, two-arm, patient-level randomized and controlled comparison of two health coaching services, one of which is already commissioned by Salford Clinical Commissioning Group and provided in the NHS by Salford Royal NHS Foundation Trust (i.e. IGR2). As a comparison of ‘active’ interventions, the expected differences in effectiveness are likely to be small, and the trial is not designed primarily to assess differences in clinical outcomes. Therefore, the objective of this trial is primarily to assess the acceptability of IGR3 compared with IGR2, with a secondary aim of examining whether IGR3 will result in a more efficient delivery method.

This trial will test the hypothesis that a web-enhanced telephone health coaching intervention for people with impaired glucose regulation (IGR3) will be more acceptable than an existing telephone-only health coaching intervention (IGR2). We are also going to examine whether IGR3 will provide a more efficient delivery method.

### Primary objective

To assess, quantitatively, whether a web-enhanced telephone coaching intervention (IGR3) is more acceptable than an existing telephone-only coaching intervention (IGR2) for people with impaired glucose regulation.

### Secondary objectives

1. To determine whether the delivery of the IGR3 intervention is more efficient than the existing commissioned IGR2 while maintaining the quality of service on a similar level
2. To explore the cost-effectiveness of IGR3 in comparison with IGR2
3. To explore and compare user and provider experience of IGR3 and IGR2 interventions qualitatively
4. To explore the impact, if any, of IGR3 compared with IGR2 on clinical outcomes relevant to diabetes prevention in people with impaired glucose regulation

### Study setting

This trial will be a single-centre study conducted in Salford, UK. Salford is a city in the north west of England made up of eight neighbourhoods with a population of 247,000 (34000 aged 65 and over) and ranked as the 16th most deprived local authority in England out of 326 [25]; approximately 14 % of the adult population are obese [26]. There are 47 general practices in the city, clustered in eight neighbourhoods.

### Interventions

**Fig. 1:**
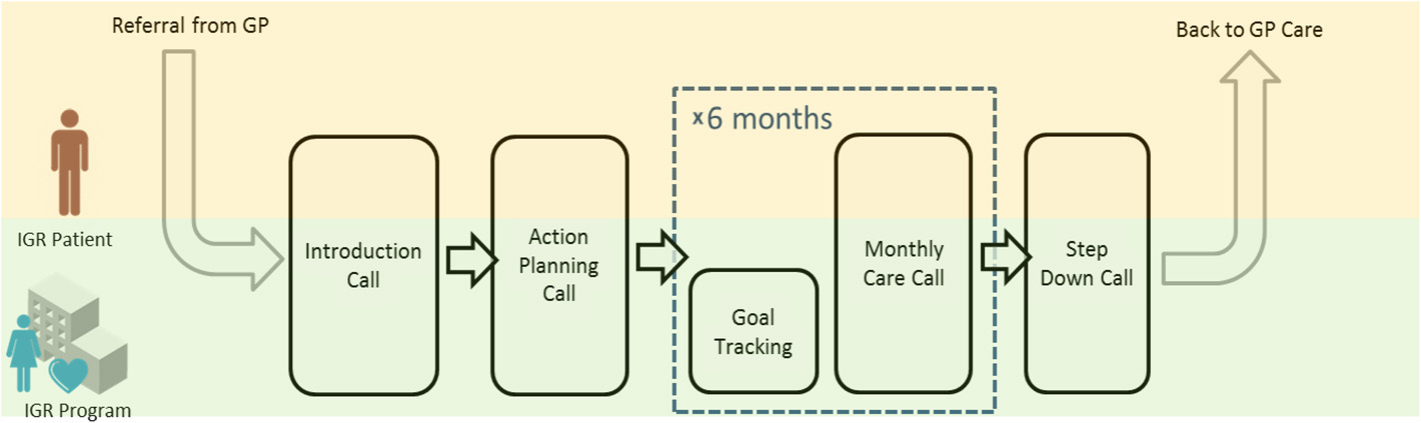
Care pathway for IGR2. GP, general practitioner; IGR, impaired glucose regulation

**Fig. 2:**
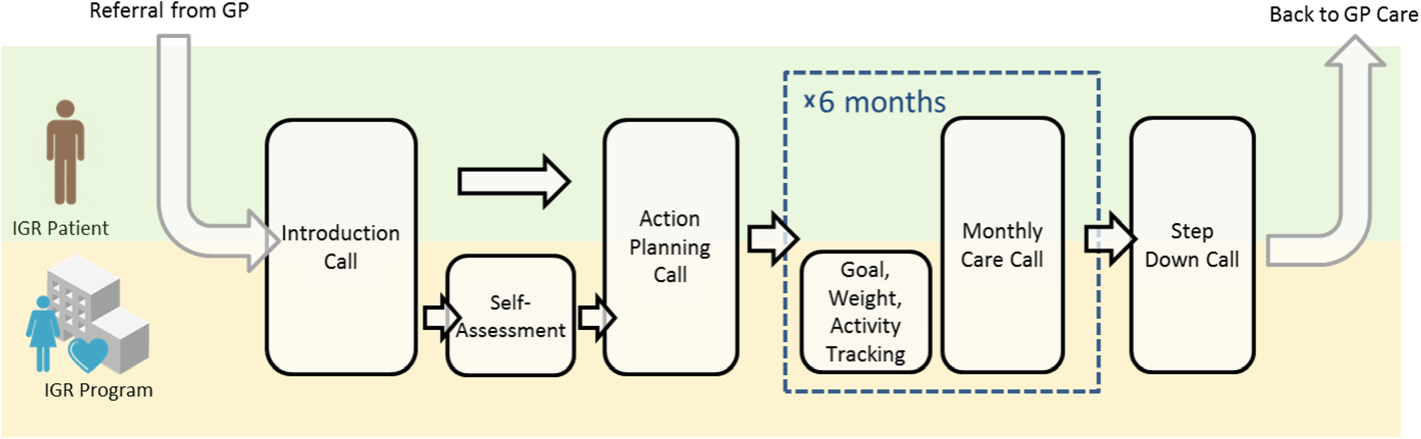
Care pathway for IGR3. GP, general practitioner; IGR, impaired glucose regulation

The intervention and control in the trial are both forms of health coaching. Figures 1 and 2 show the care pathways for IGR2 and IGR3, respectively. A comparison of the two services (highlighting their similarities and differences) is shown in Table 1. The key differences between the arms are that IGR3 provides patients with a web desktop dashboard to track progress against patient-centred goals (e.g. weight, dietary modifications) and a pedometer to monitor physical activity. Patients in the IGR3 arm also have access to educational content on the web dashboard in addition to the paper-based educational materials given to patients in the IGR2 arm.

**Table 1:** Comparison of intervention characteristics

Comparison	IGR2	IGR3
Materials	Patient information package	Web-based patient tracking system for diabetes specialist nurse
	Educational materials	Web-based patient information, videos and data recording
		SMS
		Patient information package, including pedometer, self-assessment link and log-in details
		Educational materials
Providers	Diabetes specialist nurse or dietician	Diabetes specialist nurse or dietician
	Health advisor	Health advisor
	Administrative support	Administrative support
Modes of delivery	Telephone support	Telephone support with web-based patient tracking
Location of delivery	Remote	Remote
Intervention components		
Triage	Call from diabetes specialist nurse	Call from diabetes specialist nurse
Introduction call	Call from health coach	Call from health coach
Self-assessment	Not applicable	Online self-assessment
Action planning call	Pre-call admin	Pre-call admin
	Call to patient	Call to patient
	Post-call admin	Post-call admin
Tracking	Telephone	Online and telephone
Follow-up calls 1–6	Pre-call admin	Pre-call admin
	Call to patient	Call to patient
	Post-call admin	Post-call admin
Step-down call at 9 months	As follow-up call 1	As follow-up call 1
Tailoring	Content of intervention in response to patient self-evaluation	Content of intervention in response to patient self-evaluation

### Data and outcomes

#### Demographic and clinical characteristics

Data about demographic and clinical characteristics will be entered on a case report form by the researcher at the baseline appointment. We will use sociodemographic questions from the General Practice Patient Survey [27], including sex, age, current work situation and qualifications. Ethnicity will be assessed using the 17 Census 2011 categories [28]. We will include a single-item health literacy measure, which has demonstrated good reliability and validity [29, 30], and a measure of the number and impact of long-term conditions [31].

### Primary outcome measure

#### Patient experience

Patient satisfaction will be assessed using the Client Satisfaction Questionnaire (CSQ-8), which is a generic survey instrument used widely in primary care clinical trials [32]. The CSQ-8 is an eight-item self-administered questionnaire collected at the end of service delivery and scored using a four-point Likert scale. The CSQ-8 scores range from 8 to 32, with higher values indicating higher satisfaction.

### Secondary outcome measures

#### Costs of intervention

The costs of delivery of IGR2 and IGR3 will be determined. Hitachi Europe Ltd, with support from Salford Royal Foundation NHS Trust, will provide a detailed cost breakdown of the operation of IGR2 and IGR3, including staff and infrastructure. Data on number and length of calls for each element of the care pathway in each arm will be recorded throughout the trial period. Clinicians responsible for delivery of the intervention will log call times using a standardized activity log pro-forma.

#### Health resources usage

The usage of NHS health care and social services for participants will be determined using an adapted health resources questionnaire based on our previous COINCIDE trial [33]. We will obtain information on rates of utilization of most of the major elements of health and social care through linkage with the Salford Integrated Record.

#### Health outcome measures

1. HbA1c concentration
2. Weight (kg) and body mass index
3. Quality of life: measured using the Euroqol-5D-5 L (EQ-5D-5 L) [34]. The five-item EQ-5D-5 L is a generic measure of health-related quality of life, consisting of the EQ-5D descriptive system and the EQ Visual Analogue Scale (EQ VAS). The first part consists of five domains: mobility, self-management, usual activities, pain, anxiety and depression, with five levels of severity for each domain. A utility value can then be calculated based on a population tariff. The visual analogue scale records an individual’s self-perceived health, ranging from 0 to 100.
4. Mental Health Inventory-5: this is a five-item scale that measures general mental health, including depression, anxiety, behavioural-emotional control and general positive affect [35].
5. Health experience and self-management: this will be measured by a modified version of the Summary of Diabetes Self-Care Activities. It assesses the number of days per week respondents engage in healthy and unhealthy behaviours (i.e. eating fruit and vegetable, eating red meat, undertaking exercise, drinking alcohol, and smoking) [36].
6. Patient activation: this will be measured by the Patient Activation Measure. Patient activation is a measurable outcome associated with higher quality of life, improved clinical outcomes and increase engagement with health or social care. The Patient Activation Measure is a self-report measure of patient knowledge, skills and confidence in self-management for long-term conditions [37]. We will use the short 13-item version [38].

#### Routine service level data

We will extract data related to a range of processes associated with engagement with and completion of the intervention from the secure web-based intervention hosted by North West EHealth at the University of Manchester. These data will allow us to assess patient fidelity to the pre-specified service model outlined in Table 1. Specifically, we will run queries to produce aggregate data for all patients in the IGR3 arm at the end of the intervention period related to:

1. Completeness of self-assessments
2. Number of times patients logged in to specific pages
3. Number of times patients used ‘contact advisor’ option for additional support

### Sample size

The existing IGR2 service is commissioned for 500 patients per year and we anticipate with the support of Salford Royal NHS Foundation Trust, general practitioners and diabetes specialist nurses to recruit 200 of these patients in 12 months. The trial sample size has therefore been set at 100 patients per arm, based on a pragmatic decision concerning the probable recruitment window. With an estimated 15 % attrition rate, we would have 90 % power to detect an effect on a standardized measure of 0.5 on the CSQ-8, and 70 % power to detect a standardized effect size of 0.4 with a two-sided alpha of 0.05. A significant difference in CSQ-8 scores in favour of IGR3 will prove the hypothesis that the web plus telephone health coaching intervention offers patients a better care experience than the existing telephone-only health coaching intervention.

As a comparison of two active treatments, where one is simply an enhanced version of the other, differences in clinical outcomes, quality of life and cost-effectiveness are expected to be relatively small. Therefore, the trial will not be powered to detect differences for secondary outcomes.

### Eligibility of participants

Participants will be identified from referrals into the existing Impaired Glucose Regulation Care Call service provided by Salford Royal NHS Foundation Trust. Referral criteria into the Impaired Glucose Regulation Care Call service are:

1. Moderate or high risk score on the Diabetes UK Risk score tool [39]

and

2. HbA1c = 42–47 mmol/mol (6.0–6.4 %)

or

3. Previous diagnosis of impaired glucose regulation with 1× confirmatory blood test (HbA1c within the previous 6 months).

Based on these referral criteria, the eligibility criteria for the trial recruitment are as follows.

#### Inclusion criteria

1. Aged 18 years or older
2. HbA1c between 42 and 47 mmol/mol (6.0–6.4 %) in previous 6 months
3. Access to a telephone and home internet

#### Exclusion criteria

1. Referred to the face-to-face group impaired glucose regulation education session and does not go on to receive telephone-only support
2. Diagnosis of type 2 diabetes: HbA1c of ≥48 mmol/mol (≥6.5 %)
3. Diagnosis of gestational diabetes
4. Does not read or speak English
5. Incapable of participating as indicated by general practitioner because of dementia, learning difficulties, vision or motor skills limitations, serious and enduring mental health problems

### Recruitment to the trial

General practice surgeries throughout Salford will be given promotional literature about Care Call (prepared by Hitachi Europe Ltd) to raise awareness among general practitioners about the availability of the service for people with impaired glucose regulation. All patients referred to the Care Call service will have a confirmed diagnosis of impaired glucose regulation and will have had an opportunity to discuss with their general practitioners the options available from the Care Call service. Eligible patients for the service and thus the trial will then be identified from routine contact with patients’ general practitioners.

In addition, a rapid search and find tool designed by NorthWest EHealth, FARSITE, will be used to identify further eligible patients [40]. The FARSITE software provides a safe, convenient and effective way for general practitioners to control the recruitment of their patients into clinical research, while allowing NHS-based researchers to run complex and powerful searches over anonymized population-level health record data. Because FARSITE is hosted in a secure environment located at Salford Royal NHS Foundation Trust, confidentiality of data is preserved. General practitioner data collected and processed for FARSITE are transmitted across the NHS (N3) network, using high grade encryption, by the secured local NHS data host, the General Practitioner System Supplier or Apollo Medical Systems Ltd. Patient demographics data and pseudonymized data are stored in two separated and encrypted databases. In the CATFISH study, a research nurse employed by Hitachi Europe Ltd will run FARSITE searches from general practices in Salford to generate lists of pseudonymized patient populations. The clinical teams in practice can review the selected patients, merge patient contact details using the letter generation tool and send the letters to DocMan for print and postal fulfilment services. These letters will offer patients suspected to have impaired glucose regulation to attend for a general practitioner consultation and onward referral to Care Call.

Feasibility searches using FARSITE protocols for impaired fasting glucose or impaired glucose tolerance were run in November 2014 and identified 3852 patients with suspected impaired glucose regulation. This test run showed that the FARSITE tool was capable of identifying patients with impaired glucose regulation and that there are sufficient numbers of patients with impaired glucose regulation who can be referred to the Care Call service and thus be invited to the CATFISH trial.

After triage, the diabetes specialist nurse at the Care Call service will pass details of all eligible patients to the CATFISH trial administrator. The CATFISH trial administrator will call all patients and confirm personal details (email and phone number). Each patient is given a brief overview of the CATFISH trial. The administrator will confirm that each patient meets the inclusion criteria for the CATFISH trial (access to home internet and a desktop computer or laptop), and will seek permission for the University of Manchester research team to contact them. Those patients who do not wish to be approached by researchers will be redirected to the existing Care Call service (IGR2). The contact details of those patients who do agree to be contacted will be passed to the University of Manchester research team using a secure (nhs.net) email service. Within one week, a University of Manchester researcher will then contact the patient to discuss involvement in the trial in greater detail, giving them an opportunity to ask questions about the trial.

### Participant timeline

**Fig. 3:**
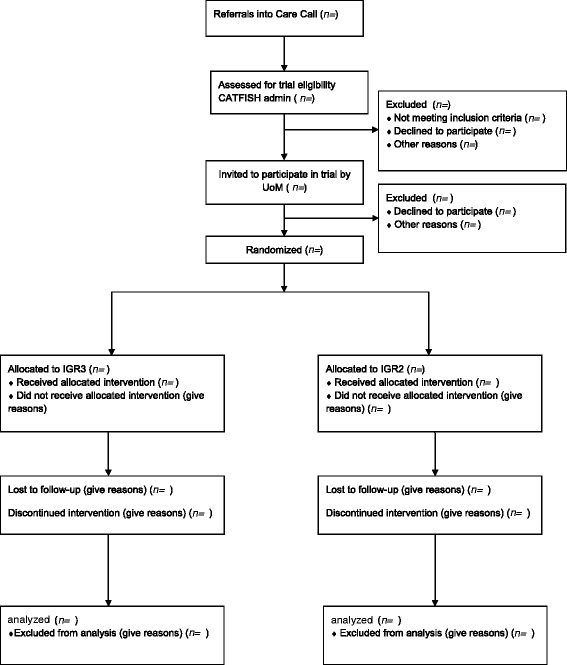
CONSORT flow diagram. UoM, University of Manchester

The recruitment window runs from July 2015 to the end of June 2016. After consenting and undertaking baseline assessments, participants will enter the IGR3 or IGR2 service, where they will receive active therapeutic contacts for 6 months, followed by a step-down call at 9 months. We will collect measures at baseline, and 9 months (i.e. 3 months after the end of the core contact period; see Table 1). Recruitment flow and timelines of assessments are shown in Fig. 3 and Table 2, respectively.

**Table 2:**
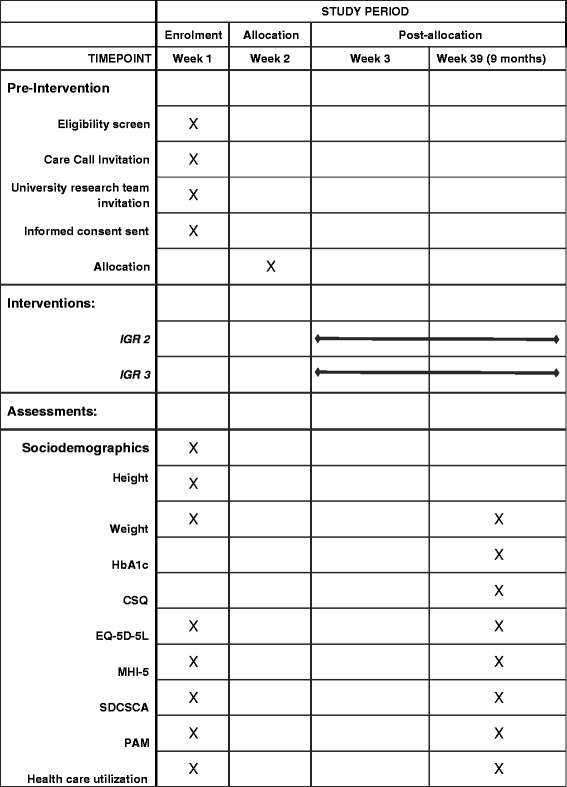
SPIRIT Schedule of enrolment, interventions and assessments

*CSQ* Client Satisfaction Measure, *EQ-5D* EuroQol five dimensions, *HbA1c* glycated haemoglobin (A1c), *MHI-5* Mental Health Inventory, *PAM* Patient Activation Measure, *SDSCA* Summary of Diabetes Self-Care Activities

### Randomization and allocation concealment

On providing consent, participants will be asked to complete baseline assessments and are then randomized using a remote and automated randomization service provided by the Manchester Academic Health Science Centre Clinical Trials Unit (MAHSC-CTU) at the Christie Hospital NHS Foundation Trust, Manchester, UK. To ensure allocation concealment, randomization will be by means of a computer-generated code implemented by a MAHSC-CTU employee and shared by telephone with the Care Call administrator following correct exchange of a password. The Care Call administrator will communicate allocations to Care Call staff (diabetes specialist nurse and health advisors). Participants will be allocated 1:1 to either IGR2 or IGR3 using minimization to ensure balance for age (<40, 40–60, >60 years) and body mass index (≤18.5, 18.6–24.9, 25.0–29.9). We will use minimization with a probability weighting of 0.75 to reduce predictability.

### Blinding

It will not be possible to blind participants to treatments but they will not be formally told which intervention is an existing service (IGR2) and which intervention is novel (IGR3). Our researchers at the University of Manchester will be informed of the MAHSC-CTU randomization number for each participant by the Care Call administrator. The MAHSC-CTU randomization number will become the primary identifier for participants in the trial. In addition to the Care Call administrator, the principal investigator (PAC) will be unmasked to allocations in the event that participants request to be unblinded. Participants will be unblinded at the end of the follow-up period, or on withdrawal from the study. The CATFISH research team at the University of Manchester will remain blind to treatment allocation until follow-up assessments have been completed. The trial statistician will remain blind to treatment allocation. However, owing to the nature of health economic analysis, it is not possible to blind the trial health economist.

### Data collection

At the baseline assessment visit, University of Manchester researchers will record the height and weight of participants and calculate body mass index using the NHS Choices body mass index healthy weight calculator [41]. Height will be measured using a Leicester stadiometer on a firm and even surface. Participants will be asked to remove their shoes and stand up straight with heels together, with heels, buttocks and shoulders pressed against the stadiometer. The University of Manchester researcher will take the measurement with the participant standing tall, looking straight ahead with the head upright and not tilted backwards.

Where participants’ cannot stand, arm span can be used as an estimate of height, using the formula: total arm span/1.06 (women) or total arm span/1.03 (men). Arm span is measured by locating and marking the edge of the right collar bone (in the sternal notch) with a pen. Participants will each be asked to place their non-dominant arm in a horizontal position. The researcher will check that the patient’s arm is horizontal and in line with the shoulders. Using a tape measure, the researcher will measure the distance from the mark on the midline at the sternal notch to the tip of the middle finger. If the arm is flat and wrist is straight, the researcher will take a reading in centimetres and repeat the process for the dominant arm to calculate the total arm span.

Weight will be measured in kilograms using Seca 875 weighing scales (Class 111 calibrated medical scales) that conform to ISO 9001:2008. Both height and weight will be recorded on a case report form. The participant’s initials is entered onto the front cover of the case report form, along with general practitioner ‘P’ code and date of completion. After height and weight have been measured, participants will be given the baseline questionnaire to complete. Researchers will be available to answer any questions the participant may have during completion. Explanation should be given without biasing the participant’s response. The researcher may also read the questions and complete the questionnaire if the participant requests this. After completing the questionnaire, the researcher will check that all questions have been attempted.

At follow-up, the researcher will contact the participant by telephone to arrange a convenient time and place to meet for the follow-up assessment. During this, call the researcher will remind the participant not to tell the researcher if they were part of the telephone-only health coaching group or the web plus telephone health coaching group. At the follow-up assessment visit, the researcher will adopt the same procedures undertaken at the baseline visit to collect and record data on height and weight. The participants will be given the follow-up questionnaire to complete and the researcher will adopt the same procedure undertaken at the baseline visit to ensure that this questionnaire is completed appropriately.

After completing follow-up assessments, participants will be invited to attend an appointment at the Clinical Research Facility at Salford Royal NHS Foundation Trust for a plasma glucose test to measure HbA1c. All blood tests will be conducted by nursing staff at the Barnes Clinical Research Facility, Salford Royal NHS Foundation Trust. Sample type and volume are fluoride oxalate (yellow), 1 ml; the reference range is 3.0–6.0 mmol/l. Laboratory staff will follow the Salford Royal NHS Foundation Trust protocol for prevention and management of potential exposure to blood-borne viruses, including needlestick and sharps injuries [42].

### Data management

After completion of blood tests and analysis, nursing staff at the Barnes Clinical Research Facility will be notified and will collect the results from the laboratory. Hard copies of the results will then be stored in a locked, secure area. A member of the CATFISH research team will visit the Barnes Clinical Research Facility at least once every two weeks to collect the results. The results will then be returned to the CATFISH office at the University of Manchester and stored securely.

Once the research team has collected the HbA1c concentration results from the Clinical Research Facility, they will be screened by the CATFISH Research Nurse. If the HbA1c concentration falls outside the normal prediabetes range expected (≥48 mmol/mol), the participant’s general practitioner will be informed by letter of the result. If the concentration remains within the prediabetes range (42–47 mmol/mol) or is within the normal range (<42 mmol/mol) the general practitioner will not be routinely informed of the result. Blood samples for HbA1c testing will be automatically archived after analysis to a secure storage unit in the pathology department at Salford Royal NHS Foundation Trust and kept at 4 °C. They will be kept for a maximum of 2 days and then sent for incineration.

Demographic and outcome data will be collected in an anonymized format using paper-based questionnaires administered face-to-face by the University of Manchester researchers. Additional data about engagement and delivery of the intervention will be captured by the secure and web-based system hosted by NorthWest EHealth at the University of Manchester. Patient confidentiality will be protected throughout all phases of data collection and analysis, in accordance with them UK Data Protection Act, 1998. The data management policy will adhere to Research Councils UK Common Principles on Data Policy and will be created by the principal investigator in accordance with the University of Manchester’s intellectual property policy and relevant third-party agreements. All paperwork will be transferred immediately to the University of Manchester and stored in a lockable fling cabinet. Paperwork with patient-identifiable data (consent form, case report form) should be stored separately from anonymized data (baseline and follow-up questionnaires).

Names and contact details of patients who decide not to take part in the trial will be destroyed by the research team. All other data collected from questionnaires after consent is given will be anonymized. University of Manchester policy on storage of personal data is 5 years after the last publication date of the study or 10 years, whichever is the greater. Consent forms will be retained as essential documents, but items such as contact details will be deleted as soon as they are no longer needed.

### Statistical analysis

We will report the trial and analysis according to CONSORT standards, including full details of use of the various telephone coaching components [43]. The data analyst will be masked to treatment allocation.

For most outcomes, we will present descriptive data on baseline and follow-up scores, to allow assessment of change in IGR2 and IGR3 patients, as well as comparison with outcomes found in pilot evaluations [16, 17]. The focus will be on assessing whether IGR3 achieves at least as good outcomes as IGR2. This will not involve a formal assessment of equivalence.

We will formally test for differences between IGR3 and IGR2 on patient experience using the CSQ-8. Analysis will follow intention-to-treat principles and a pre-specified plan. The core analysis will be via linear regression, using robust standard errors adjusted for the clustering of patients within practices. We will control for baseline values of each outcome and design factors. We will apply multiple imputation to baseline and 9 month variables with missing values by the chained equations approach using scores on all primary and secondary outcome measures (at baseline and follow-up). We will use 20 multiple imputation sets, as this will provide appropriate stability of results. Analyses will be conducted using STATA (version 14) with an alpha significance value of 5 %. We will report standardized effect sizes for all outcomes to aid comparison with published studies.

### Health economic analysis

The health economic analysis will comprised two parts, both of which will assess the cost-effectiveness of health coaching with a web-based IT platform (IGR3) compared with health coaching alone (IGR2) among people with prediabetes. The first will be an incremental cost-effectiveness analysis from a clinical commissioning group perspective using patient satisfaction and intervention costs to derive a cost per additional unit of patient satisfaction. The second analysis will be conducted from an NHS and personal social services perspective [44]. Costs will include intervention costs and healthcare and social services resource costs. The quantity of resource use will be collected by questionnaire and a set of national average unit costs will be applied (e.g. [45]). The use of the EQ-5D-5 L will enable the estimation of quality-adjusted life years by calculating the area under the curve [46]. An incremental cost-effectiveness ratio (cost per additional quality-adjusted life year) will be used to assess cost-effectiveness of IGR3 in comparison with IGR2. Cost-effectiveness planes and cost-effectiveness acceptability curves will be constructed to reflect any uncertainty in the results and threshold.

### Qualitative study

Process evaluations of complex interventions can be used to explain outcomes through an evaluation of how casual assumptions about how an intervention might work are related to the way it was implemented and how it produces change within particular contexts. Drawing on guidance from the Medical Research Council, a focus on understanding implementation (the what and how of intervention delivery), mechanisms of impact (pathways to change), and contextual factors can inform the design and conduct of a process evaluation [47]. However, the framework proposed by the Medical Research Council is not easily operationalized in the absence of a programme theory set out as a logic model. Programme theory articulates the hypothesized connections between the programme components and the outcomes to be assessed and is often underpinned by a theory of change [48]. Logic models offer a visual way of representing the ‘if… then…’ relationships between the resources needed to deliver the programme, the activities planned and their outputs, and the intended results of the programme. Using programme theory to drive the evaluation can help differentiate between programme theory failure, i.e. whether the intervention failed because of weaknesses in the underlying theory of change, and programme implementation failure, i.e. whether the intervention failed because of weaknesses in the way it was delivered [49].

**Fig. 4:**
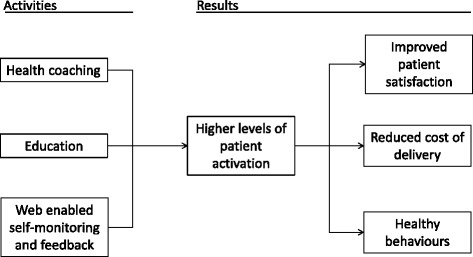
Theory of change model in CATFISH

In this trial, the theory of change presupposes that health coaching supported by web-enabled self-monitoring and feedback and education will increase patient activation, which, in turn, will result in increased patient satisfaction, reductions in the cost of service delivery and positive changes in health behaviours known to delay or prevent type 2 diabetes (Fig. 4). As such, greater effects are anticipated among participants in the web plus telephone coaching group than in the telephone-only group.

At the heart of this model is the concept of patient activation, which captures key ingredients known to predict patients’ capacity to engage in self-managing their health and use of healthcare: knowledge, skill and confidence [38]. Higher patient activation has been shown to predict engagement in preventive behaviours, such as attending regular check-ups, and healthy behaviours, such as regular exercise, treatment adherence and self-monitoring [50]. Moreover, activated patients are more likely to have clinical outcomes, such as HbA1c concentration and body mass index in the normal range [51]. Critically, highly activated patients are more satisfied with their care experience and have lower rates of hospital admissions and emergency room use, possibly leading to reductions in the cost of their care [52, 53].

**Fig. 5:**
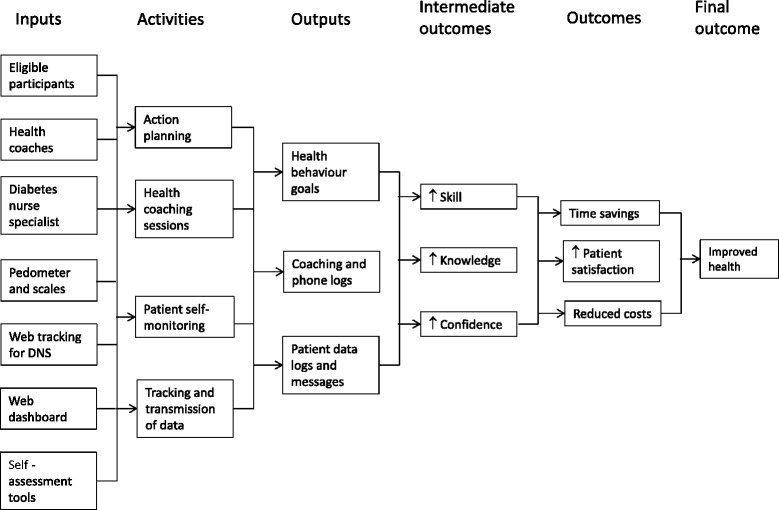
Logic model of programme components in CATFISH. DNS, domain name system

Taking this theory of change as a starting point, Fig. 5 shows the logic model for the web plus telephone health coaching intervention tested in this trial. It is read from left to right, and includes a detailed breakdown of the resources and activities associated with delivering the intervention, along with details of the anticipated results, which include outputs, outcomes and impact over time. This logic model will facilitate qualitative evaluation of key programme vantage points related to context, implementation and outcomes [54]. While this trial is not a formal test of clinical effectiveness, qualitative evidence drawn from patient participants and also from health professionals engaged in delivery of the interventions will strengthen our understanding about user experience and impact of what was delivered, leading to greater opportunities to report about how the interventions might work in comparable and different contexts. Specific to the evaluation of IGR3, the evaluation will also be informed by evidence about dynamic factors that moderate individual acceptance of IT [55].

#### Qualitative data collection

Qualitative assessments using semi-structured interviews with patient participants drawn from both arms of the trial will take place after quantitative follow-up data have been collected. Semi-structured interviews offer opportunities to cover, in-depth, a range of topics relevant to the research questions, but also allow for exploration and probing of issues raised during the interview. In this trial, we will assess patient experience of IGR3 compared with IGR2, with a focus on understanding whether the web enhancements led to greater levels of activation and thus greater engagement with managing their health. We will also capture data from health professionals about the experience of implementing web or telephone health coaching in the context of impaired glucose regulation, with a focus on understanding acceptability and feasibility of using web platforms to enhance patient engagement in action planning and behaviour change.

Purposeful maximum variation sampling will be used to identify patient participants sampled for age, baseline body mass index and intervention arm. All interviews will be conducted before outcome analysis is complete, to allow for *a-priori* exploration of user acceptance and experience of implementation. We will aim to conduct approximately 40 interviews in total, comprising approximately 20 participants drawn from both arms of the trial. Where feasible, professionals engaged in the commissioning, management and delivery of the health coaching service will also be interviewed. Up to 15 professional interviews will be conducted.

#### Qualitative data analysis

Interviews will be transcribed verbatim and analyzed thematically using standard approaches informed by Framework [56]. There are five key stages in this type of analysis:

1. Familiarization – the transcripts will be read thoroughly by all researchers to identify key themes.
2. Developing a thematic framework – a framework will be developed that will be applied to the transcripts. Following discussions with co-researchers, this framework will then be expanded and refined.
3. Indexing – themes and emerging sub themes will be labelled and indexed.
4. Charting – framework involves devising a series of thematic charts or matrices.
5. Mapping and interpretation – the aim is to bring out the key characteristics and map and interpret the data as a whole.

A benefit of using Framework analysis is that strategies and recommendations for practice and policy may be elicited at an early stage.

### Data monitoring

The trial will be supervised independently by members of the trial steering committee. This committee will meet twice during the active recruitment period and has responsibility for monitoring progress of the trial, adherence to the protocol, patient safety and consideration of new information. Membership includes the principal investigator (PAC), the chairperson (Professor Christie Deaton, University of Cambridge, UK), and two other independent members (Dr Barbara Barett, King’s College London, UK and Dr Daniel Hind, University of Sheffield, UK). The trial statistician will attend when appropriate. At the chair’s discretion, an observer from Hitachi Europe Ltd will attend the trial steering committee. Given the nature of this trial it is unlikely that there are critical patient safety issues for a separate data monitoring and ethics committee to consider and there will be no formal stopping rules. Terms of reference of the trial steering committee are available on request from the principal investigator.

### Adverse event reporting

An adverse event is any untoward and unexpected medical occurrence in a patient or clinical study subject. Adverse events are likely to be rare but should be reported to the principal investigator by the researcher or Care Call team. Although CATFISH is a trial of a non-investigational medicinal product, serious adverse events that are both related to the research procedures and are unexpected should be reported immediately (within 24 hours) either orally or in writing to the research sponsor (University of Manchester). This immediate report will be followed by a detailed written report sent to the NHS Research Ethics Committee that granted approval for the trial (East of England – Cambridgeshire and Hertfordshire) within 15 days of the study investigator becoming aware of the event.

### Research ethics

The trial will be conducted in accordance with the UK Department of Health’s Research Governance Framework in health and social care and adhere to the ethical principles of the Helsinki Declaration [57]. All research staff involved in the conduct of the trial will meet the standards laid out in the *ICH Harmonised Tripartite Guideline for Good Clinical Practice* [58]. All participants will be offered a high street voucher worth £20 after completing baseline and the follow-up assessments.

### Amendments

Substantial amendments will be communicated to the NHS Research Ethics Committee for the East of England (Cambridgeshire and Hertfordshire), following the process outlined by the NHS Health Research Authority. Since starting the trial one substantial amendment has been submitted and granted. This related to permission for a promotional flyer to be used in general practice to promote the Impaired Glucose Regulation Care Call service among general practitioners. This amendment also included provision for the eligibility criteria to be changed to bring the trial into line with evaluation parameters proposed by the NHS National Diabetes Prevention Programme, i.e. only patients with a HbA1c concentration between 42 and 47 mmol/mol in the previous 6 months can be referred into the Care Call service and subsequently invited to take part in the trial.

### Access to data and dissemination policy

As the sponsor of the trial, the University of Manchester will remain the custodians of the data collected from participants during the trial and will not share data with any third party, including private companies, without the consent of the participants. Data will not be released to third parties or private companies (including the funder) before the trial has been completed and will be analyzed by an independent evaluation team at the University of Manchester. No interim analysis is planned and no interim data will be shared with the funder or third parties. In recognition of the importance of transparency and need to increase trust in clinical trial results both Hitachi Europe Ltd, and the University of Manchester will agree to data sharing in accordance with proposals outlined by the International Committee of Medical Journal Editors, which state that authors share with others the deidentified individual-patient data underlying the results presented in the trial report (including tables, figures, and appendices or supplementary material) no later than 6 months after publication [59]. Hitachi, as the funder of the trial and as owners of the technology to be tested, may wish to invoke a brief embargo (up to 3 months) on data sharing before publication in an open-access journal.

## Discussion

Interventions that rely solely on telephone contact (with no self-monitoring of blood glucose) are no more effective than standard care in improving glycaemic control [60], signalling an opportunity to further develop and evaluate interventions that combine telephone interventions with self-monitoring and electronic transfer of data between patients and healthcare providers. Before embarking on expensive and time-consuming trial assessment of clinical effectiveness and cost-effectiveness, however, a key challenge is to understand from the patient perspective if enhancing telephone health coaching interventions with IT platforms improves acceptability and usability and thereby engagement in diabetes prevention programmes. This trial will offer important insights about whether a web-based IT platform can enhance engagement in a telephone health coaching intervention provided by health advisors for people with impaired glucose regulation. The results will have implications for the design of future definitive cost-effectiveness trials in settings where there is no existing diabetes prevention programme.

The trial design is potentially limited by restrictions on participant recruitment. Currently, referral into the Care Call service at Salford Royal NHS Foundation Trust is heavily reliant on general practice and there are limited routes into the service from other sources in the community, e.g. pharmacy and public health. Participants in the CATFISH trial will therefore primarily be drawn from patients already ‘in the system’ and known to general practitioners and may have been given behaviour change advice in the past. Additionally, achieving our recruitment target may be jeopardized by only sourcing participants for the CATFISH trial from referrals into the Care Call service – recruitment into the trial will be contingent on sufficient throughput in the clinical service. However, Salford Royal NHS Foundation Trust is one of seven demonstrator sites for the NHS National Diabetes Prevention Programme in the NHS and there is an ongoing commitment to increase capacity in the Care Call service, with a likely benefit for the CATFISH trial. Furthermore, the Care Call service attracts referrals from all eight neighbourhoods across Salford, increasing opportunities to recruit participants with different socio-economic profiles. The design and recruitment strategy are thus pragmatic and thus more likely to be generalizable.

Working with industry partners in health services research is novel and presents unique challenges, not least the need to maintain independence. Hitachi Europe Ltd, have invested in the Care Call service at Salford Royal NHS Foundation Trust to improve efficiencies, such as employing an administrator and a research nurse to expedite quality referrals into the service. With the support of the trial steering committee, the CATFISH trial is being run as an independent evaluation and we have ensured that sufficient safeguards are in place to preserve independence throughout all phases of the trial. This includes a commitment to open-access publication and data sharing, in keeping with policies to prevent publication and outcome reporting bias of clinical trials.

## Trial status

Trial registration was initiated prospectively with ISRCTN on 23 April 2015. Failure of administrative functions on the part of BioMed Central who host and curate ISRCTN and the University of Manchester led to delay in payment and registration was not finalised until 1 July 2015. The recruitment started on 30 June 2015, after the initiation of public registration.

Recruitment to the trial began in June 2015 and completed in May 2016. A total of 209 participants were recruited and randomized. The trial is now in follow-up and data collection will be completed at the latest in March 2017; results will be available in May 2017.

## Abbreviations

CONSORT, Consolidated Standards of Reporting Trials; CSQ-8, Client Satisfaction Questionnaire; EQ-5D, Euroqol-5D; HbA1c, Glycated haemoglobin; IT, information technology; MAHSC-CTU, Manchester Academic Health Science Centre Clinical Trials Unit; NHS, National Health Service (UK); NICE, National Institute for Health and Care Excellence; SMS, short message service; SPIRIT, Standard Protocol Items: Recommendations for Interventional Trials

## Additional file

Additional file 1: SPIRIT 2013 Checklist. (DOC 121 kb)

## References

[1] American Diabetes Association (2009). Diagnosis and classification of diabetes mellitus. Diabetes Care.

[2] Diabetes UK (2015). Facts and stats.

[3] Fowler MJ (2008). Microvascular and macrovascular complications of diabetes. Clin Diabetes.

[4] Hex N, Bartlett C, Wright D, Taylor M, Varley D (2012). Estimating the current and future costs of type 1 and type 2 diabetes in the UK, including direct health costs and indirect societal and productivity costs. Diabet Med.

[5] Health and Social Care Information Centre (2014). Statistics on obesity, physical activity and diet: England 2014.

[6] Bombelli M, Facchetti R, Sega R, Carugo S, Fodri D, Brambilla G, Giannattasio C, Grassi G, Mancia G (2011). Impact of body mass index and waist circumference on the long-term risk of diabetes mellitus, hypertension, and cardiac organ damage. Hypertension.

[7] Santaguida PL BC, Hunt D, Morrison K, Gerstein H, Raina P, Booker L, Yazdi H (2005). Diagnosis, prognosis, and treatment of impaired glucose tolerance and impaired fasting glucose. Evidence Report/Technology Assessment No 128.

[8] Public Health England (2015). A systematic review and meta-analysis assessing the effectiveness of pragmatic lifestyle interventions for the prevention of type 2 diabetes mellitus in routine practice.

[9] Palmer S, Tubbs I, Whybrow A (2003). Health coaching to facilitate the promotion of healthy behaviour and achievement of health-related goals. Int J Health Promot Educ.

[10] McLean S, Protti D, Sheikh A (2011). Telehealthcare for long term conditions. BMJ.

[11] Kivela K, Elo S, Kyngas H, Kaariainen M (2014). The effects of health coaching on adult patients with chronic diseases: a systematic review. Patient Educ Couns.

[12] McLean S, Sheikh A, Cresswell K, Nurmatov U, Mukherjee M, Hemmi A, Pagliari C (2013). The impact of telehealthcare on the quality and safety of care: a systematic overview. PLoS One.

[13] Hutchison AJ, Breckon JD (2011). A review of telephone coaching services for people with long-term conditions. J Telemed Telecare.

[14] Steventon A, Tunkel S, Blunt I, Bardsley M (2013). Effect of telephone health coaching (Birmingham OwnHealth) on hospital use and associated costs: cohort study with matched controls. BMJ.

[15] Young RJ, Taylor J, Friede T, Hollis S, Mason JM, Lee P, Burns E, Long AF, Gambling T, New JP (2005). Pro-active call center treatment support (PACCTS) to improve glucose control in type 2 diabetes: a randomized controlled trial. Diabetes Care.

[16] Savas LA, Grady K, Cotterill S, Summers L, Boaden R, Gibson JM (2015). Prioritising prevention: implementation of IGT Care Call, a telephone based service for people at risk of developing type 2 diabetes. Prim Care Diabetes.

[17] Savas LA, Grady K. The IGR Care Call project evaluation report. Manchester: University of Manchester; 2013. http://clahrc-gm.nihr.ac.uk/wp-content/uploads/Final-IGR-report-Jan-2014.pdf. Accessed 03 Aug 16.

[18] Sundiatu DSG, Thomas P, Angela S (2012). Changing patient behavior: the next frontier in healthcare value. Health Int.

[19] Small N, Bower P, Chew-Graham CA, Whalley D, Protheroe J (2013). Patient empowerment in long-term conditions: development and preliminary testing of a new measure. BMC Health Serv Res.

[20] Norman GJ, Zabinski MF, Adams MA, Rosenberg DE, Yaroch AL, Atienza AA (2007). A review of eHealth interventions for physical activity and dietary behavior change. Am J Prev Med.

[21] El-Gayar O, Timsina P, Nawar N, Eid W (2013). A systematic review of IT for diabetes self-management: are we there yet?. Int J Med Inform.

[22] Sawesi S, Rashrash M, Phalakornkule K, Carpenter JS, Jones JF (2016). The impact of information technology on patient engagement and health behavior change: a systematic review of the literature. JMIR Med Inform.

[23] Chan AW, Tetzlaff JM, Gotzsche PC, Altman DG, Mann H, Berlin JA, Dickersin K, Hrobjartsson A, Schulz KF, Parulekar WR (2013). SPIRIT 2013 explanation and elaboration: guidance for protocols of clinical trials. BMJ.

[24] Chan AW, Tetzlaff JM, Altman DG, Laupacis A, Gotzsche PC, Krleza-Jeric K, Hrobjartsson A, Mann H, Dickersin K, Berlin JA (2013). SPIRIT 2013 statement: defining standard protocol items for clinical trials. Ann Intern Med.

[25] Department for Communities and Local Government (2015). The English indices of deprivation 2015.

[26] Salford Primary Care Trust. Refreshed strategic plan 2009–2014. www.ibyd.co.uk/bhf/documents/Salford_PCT/StrategicPlan2009-2014.pdf. Accessed 03 Aug 16.

[27] Campbell J, Smith P, Nissen S, Bower P, Elliott M, Roland M (2009). The GP patient survey for use in primary care in the National Health Service in the UK – development and psychometric characteristics. BMC Fam Pract.

[28] Office for National Statistics (2015). Harmonised Concepts and Questions for Social Data Sources. Primary Principles. Ethnic Group. Version 3.3.

[29] Morris NS, MacLean CD, Chew LD, Littenberg B (2006). The Single Item Literacy Screener: evaluation of a brief instrument to identify limited reading ability. BMC Fam Pract.

[30] Jeppesen KM, Coyle JD, Miser WF (2009). Screening questions to predict limited health literacy: a cross-sectional study of patients with diabetes mellitus. Ann Fam Med.

[31] Bayliss EA, Ellis JL, Steiner JF (2009). Seniors’ self-reported multimorbidity captured biopsychosocial factors not incorporated into two other data-based morbidity measures. J Clin Epidemiology.

[32] Attkisson CC, Zwick R (1982). The Client Satisfaction Questionnaire: psychometric properties and correlations with service utilization and psychotherapy outcome. Eval Program Plann.

[33] Coventry P, Lovell K, Dickens C, Bower P, Chew-Graham C, McElvenny D, Hann M, Cherrington A, Garrett C, Gibbons CJ (2015). Integrated primary care for patients with mental and physical multimorbidity: cluster randomised controlled trial of collaborative care for patients with depression comorbid with diabetes or cardiovascular disease. BMJ.

[34] Herdman M, Gudex C, Lloyd A, Janssen M, Kind P, Parkin D, Bonsel G, Badia X (2011). Development and preliminary testing of the new five-level version of EQ-5D (EQ-5D-5 L). Qual Life Res.

[35] Berwick DM, Murphy JM, Goldman PA, Ware JE, Barsky AJ, Weinstein MC (1991). Performance of a five-item mental health screening test. Med Care.

[36] Toobert DJ, Hampson SE, Glasgow RE (2000). The summary of diabetes self-care activities measure: results from 7 studies and a revised scale. Diabetes Care.

[37] Hibbard JH, Stockard J, Mahoney ER, Tusler M (2004). Development of the Patient Activation Measure (PAM): conceptualizing and measuring activation in patients and consumers. Health Serv Res.

[38] Hibbard JH, Mahoney ER, Stockard J, Tusler M (2005). Development and testing of a short form of the Patient Activation Measure. Health Serv Res.

[39] Diabetes UK. Diabetes risk score assessment tool. 2016. https://www.diabetes.org.uk/professionals/diabetes-risk-score-assessment-tool/. Accessed 03 Aug 16.

[40] NorthWest EHealth. FARSITE. 2014. http://nweh.co.uk/products/farsite. Accessed 03 Aug 16.

[41] NHS Choices. BMI healthy weight calculator. 2015. http://www.nhs.uk/Tools/Pages/Healthyweightcalculator.aspx. Accessed 03 Aug 16.

[42] Salford Royal NHS Foundation Trust (2015). Prevention and management of potential exposure to blood borne viruses including needlestick and sharps injuries. Issue No. 4.

[43] Schulz KF, Altman DG, Moher D (2010). CONSORT 2010 statement: updated guidelines for reporting parallel group randomised trials. BMJ.

[44] National Institute for Health and Care Excellence (NICE) (2013). Guide to the methods of technology appraisal 2013.

[45] Curtis L (2013). Unit costs of health and social care 2013.

[46] Richardson G, Manca A (2004). Calculation of quality adjusted life years in the published literature: a review of methodology and transparency. Health Econ.

[47] Moore GF, Audrey S, Barker M, Bond L, Bonell C, Hardeman W, Moore L, O’Cathain A, Tinati T, Wight D (2015). Process evaluation of complex interventions: Medical Research Council guidance. BMJ.

[48] Knowlton LW, Phillips CC (2013). The logic model guidebook. better strategies for great results.

[49] Bickman L (1987). Using program theory in evaluation.

[50] Hibbard JH, Gilburt H (2014). Supporting people to manage their health. An introduction to patient activation.

[51] Greene J, Hibbard JH (2012). Why does patient activation matter? An examination of the relationships between patient activation and health-related outcomes. J Gen Intern Med.

[52] Hibbard JH, Greene J (2013). What the evidence shows about patient activation: better health outcomes and care experiences; fewer data on costs. Health Aff (Millwood).

[53] Kinney RL, Lemon SC, Person SD, Pagoto SL, Saczynski JS (2015). The association between patient activation and medication adherence, hospitalization, and emergency room utilization in patients with chronic illnesses: a systematic review. Patient Educ Couns.

[54] WK Kellogg Foundation (2004). Using logic models to bring together planning, evaluation, and action. Logic Model Development Guide.

[55] Venkatesh VMM, Davis GB, Davis FD. User acceptance of information technology: toward a unified view. MIS Q. 2003; n27:425–78.

[56] Ritchie J, Spencer L, Bryman A, Burgess RG (1994). Qualitative data analysis for applied policy research. Analyzing qualitative data.

[57] World Medical Association General Assembly (2008). World Medical Association Declaration of Helsinki: ethical principles for medical research involving human subjects – Seoul amendment.

[58] Medical Research Council (1998). Guidelines for good clinical practice in clinical trials.

[59] Taichman DB, Backus J, Baethge C, Bauchner H, de Leeuw PW, Drazen JM, Fletcher J, Frizelle FA, Groves T, Haileamlak A (2016). Sharing clinical trial data – a proposal from the International Committee of Medical Journal Editors. N Engl J Med.

[60] Suksomboon N, Poolsup N, Nge YL (2014). Impact of phone call intervention on glycemic control in diabetes patients: a systematic review and meta-analysis of randomized. controlled trials. PLoS One.

